# Variability in Maturity, Oil and Protein Concentration, and Genetic Distinctness among Soybean Accessions Conserved at Plant Gene Resources of Canada

**DOI:** 10.3390/plants11243525

**Published:** 2022-12-14

**Authors:** Yong-Bi Fu, Elroy R. Cober, Malcolm J. Morrison, Frédéric Marsolais, Rong Zhou, Ning Xu, A. Claire Gahagan, Carolee Horbach

**Affiliations:** 1 Plant Gene Resources of Canada, Saskatoon Research and Development Centre, Agriculture and Agri-Food Canada, 107 Science Place, Saskatoon, SK S7N 0X2, Canada; 2 Ottawa Research and Development Centre, Agriculture and Agri-Food Canada, Ottawa, ON K1A 0C6, Canada; 3 Genomics and Biotechnology, London Research and Development Centre, Agriculture and Agri-Food Canada, London, ON N5V 4T3, Canada; 4 Saskatoon Research and Development Centre, Agriculture and Agri-Food Canada, 107 Science Place, Saskatoon, SK S7N 0X2, Canada

**Keywords:** soybean germplasm, maturity, oil and protein concentration, genetic diversity, core subset, plant germplasm conservation

## Abstract

Soybean (*Glycine max* (L.) Merr.) is one of the important crops in Canada and has the potential to expand its production further north into the Canadian Prairies. Such expansion, however, requires the search for adapted soybean germplasm useful for the development of productive cultivars with earlier maturity and increased protein concentration. We initiated several research activities to characterize 848 accessions of the soybean collection conserved at Plant Gene Resources of Canada (PGRC) for maturity, oil and protein concentration, and genetic distinctness. The characterization revealed a wide range of variations present in each assessed trait among the PGRC soybean accessions. The trait variabilities allowed for the identification of four core subsets of 35 PGRC soybean accessions, each specifically targeted for early maturity for growing in Saskatoon and Ottawa, and for high oil and protein concentration. The two early maturity core subsets for Saskatoon and Ottawa displayed days to maturity ranging from 103 to 126 days and 94 to 102 days, respectively. The two core subsets for high oil and protein concentration showed the highest oil and protein concentration from 25.0 to 22.7% and from 52.8 to 46.7%, respectively. However, these core subsets did not differ significantly in genetic distinctness (as measured with 19,898 SNP markers across 20 soybean chromosomes) from the whole PGRC soybean collection. These findings are useful, particularly for the management and utilization of the conserved soybean germplasm.

## 1. Introduction

Germplasm evaluation and characterization have played an important role in the management and utilization of germplasm conserved in genebanks worldwide [1,2]. Many core sets of germplasm have been developed from large germplasm collections [3,4,5,6]. These representative accessions have facilitated germplasm management and enhanced germplasm utilization [7,8,9,10]. However, many collections are not adequately evaluated and characterized, as evaluation and characterization efforts require substantial resources and funding support [11,12]. Many conserved germplasm accessions are recorded only with basic germplasm descriptors such as passport data and the accession-level information on conserved germplasm is largely lacking [13,14]. This problem is one of the major restraints to wider germplasm utilization [2,15]. 

Soybean (*Glycine max* (L.) Merr.) is one of the most important crops in Canada [16] with 6.27 million tonnes produced in 2021 [17]. There is potential to expand its production further north into the Canadian Prairies [18,19,20]. Such expansion, however, requires the search for adapted soybean germplasm useful for the development of productive cultivars with earlier maturity and increased protein concentration [21]. Plant Gene Resources of Canada (PGRC; the Canadian national seed genebank at Saskatoon) maintains a soybean germplasm collection of 1031 accessions. These accessions were largely collected from Canadian soybean breeding programs during the last 50 years and acquired from the USDA–ARS soybean collection and the N.I. Vavilov All-Russian Institute of Plant Genetic Resources during the last 15 years as accessions with known early maturity. Characterization of this soybean collection should facilitate the germplasm search for useful short-season soybean materials for effective cultivar development. 

We have initiated several research activities to characterize the PGRC soybean collection since 2017. The first effort was to apply a genotyping-by-sequencing technique to genotype 541 PGRC soybean accessions and 30 Ottawa breeding lines, and to identify accessions with the most genetic distinctness and redundancy. Major findings from this effort have been published [22]. The second trial was to evaluate the early maturity of 156 accessions in the field in Saskatoon and Ottawa. The third activity was to screen 776 accessions for oil and protein concentration. These research efforts have generated the first large set of characterization data on the PGRC soybean germplasm in maturity, oil and protein concentration, and genetic distinctness. Analyses were also made on these characterization data with the aim to develop trait-specific core subsets to enhance germplasm management and utilization. This paper was written to report the patterns of variability in the assessed traits and four trait-specific core subsets of soybean germplasm. 

## 2. Materials and Method

There were only 824 accessions of the PGRC soybean collection used for the germplasm characterizations described in this paper (Table S1), as many accessions in the collection had insufficient seeds for distribution. Additionally, different sets of accessions varying in size from 131 to 776 were employed for different characterizations, largely due to the availability of research resources.

### 2.1. Analysis of Early Maturity

The field trial of early maturity started with a greenhouse seed increase. We selected 131 accessions representing 19 countries of origin and one group of unknown origin from the PGRC soybean collection. We also acquired 30 short-season soybean cultivars and breeding lines developed and released from the Ottawa soybean breeding program of Agriculture and Agri-Food Canada (AAFC) [23,24] (Elroy R. Cober, personal communication). The seed increase was conducted in the Saskatoon Research and Development Centre greenhouse. For each accession, three eight-inch pots were planted with five seeds in each pot. Plants were grown in 16 h of daylight with a day temperature of 22 °C and a night temperature of 16 °C. Plants were grown to maturity and the seed was collected.

The greenhouse increase generated 146 accessions (including 30 from Ottawa) with sufficient seeds for the field trials. Ten additional accessions of known photoperiod response were also acquired from the Ottawa soybean breeding program (Elroy R. Cober, personal communication) and used as checks. The trials were conducted in the farm fields of Saskatoon Research and Development Centre (52°09′15″ N, 106°34′33″ W and 510 m ASL) and Ottawa Research and Development Centre (45°23′24.5″ N, 75°42′55.6″ W and 79 m ASL). At both locations, samples were randomized and seeded by hand in one-meter single-row plots with two replications. Twenty-five seeds were planted per row, with four-centimetre spacing between seeds. Rows were spaced 30 to 40 cm apart with a one- or two-meter pathway between plots. Seeding in Saskatoon took place on 22 May 2018, while seeding in Ottawa was on 24–25 May 2018. Phenological progress was measured using the Fehr and Caviness scale [25]. Each stage was recorded when 50% of the plants in a row achieved that stage. Flower colour, leaf shape, and pubescence colour were also documented. Accessions that reached maturity were harvested by hand and threshed. After threshing, harvest seed weight was measured and seed characteristics (seed coat colour, hilum colour, seed splitting, and seed coat texture) were recorded for all surviving accessions.

### 2.2. Analysis of Seed Oil and Protein Concentration

We acquired seeds of 776 PGRC soybean accessions to analyze seed oil and protein concentration. Three samples each with seven seeds were randomly selected without replacement from each accession envelope for seed oil analysis, and two samples of each accession used for oil analysis were randomly selected later for seed protein analysis. However, there were 25 samples each with 6 seeds for 20 accessions and 4 samples each with 6 seeds for 4 accessions for oil and protein analyses, respectively, due to the acquisition of an insufficient amount of seeds for those accessions. 

The dry seed oil concentration of each sample was determined by time-domain nuclear magnetic resonance spectroscopy (TD-NMR) [26,27] using a Bruker minispec mq10 NMR Analyzer (Bruker, Billerica, MA, USA). The instrument was calibrated with canola seed samples extracted with hexane. Oil and moisture concentration was measured for each sample that had been dried for 48 h at 40 °C. The sample was weighed and placed in a 13 mm NMR tube for measurement. Results were expressed as a percentage on a whole-seed dry-matter (zero moisture) basis and averaged over three samples for each accession. 

The seed protein concentration of each sample was determined using the combustion nitrogen analysis method [28] with a LECO FP-528 Nitrogen/Protein Determinator (LECO Corporation, St. Joseph, MI, USA). EDTA (EDTALCRM^®^ Certified Reference Material, LECO Corporation, St. Joseph, MI, USA) was used to calibrate nitrogen concentration. Before analysis, each sample was prepared by grinding 6–7 seeds to a powder using a Wiley^®^ Mini-Mill with a 0.425 mm (US Std #40) mesh screen (Thomas Scientific, Swedesboro, NJ, USA). The total percent nitrogen was measured from a 150–250 mg subsample. Protein concentration was calculated by multiplying the nitrogen concentration (%) by a factor of 6.25 (AOCS 1997). Results were averaged over two samples for each of the 776 accessions. 

### 2.3. Analysis of Genetic Distinctness

We genotyped 541 PGRC soybean accessions and 30 Ottawa breeding lines using a genotyping-by-sequencing technique. The accession selection, DNA extraction, and library development for sequencing, bioinformatics analysis of sequence data, and analysis of genetic distinctness were described in detail in the published paper [22]. For this paper, we extracted the published data of genetic distinctness for the assayed accessions from Table S2 of Fu et al. [22] and analyzed its variability and correlations with early maturity and seed oil and protein concentration to support the development of trait-specific core subsets with a broader genetic base. 

Briefly, the genetic distinctness of a soybean accession is defined as the average pairwise dissimilarity (APD) of the accession sample against the rest of the accession samples based on the genotyping of 19,898 SNP markers across 20 soybean chromosomes, as developed by Fu [29]. The higher the APD value, the more genetically distinct the sample is in the collection. The lower the APD value, the more genetically redundant the sample is in the collection. Ranking the APD values of all the assayed samples provides a means for identifying the most distinct and redundant samples [29]. 

### 2.4. Statistical Analysis

The collected data of maturity and oil and protein concentration were first subjected to two-factor ANOVA analysis using an R script written with the dplyr package [30] to determine the statistical significance of field site and replication. Similarly, one-factor ANOVA was also performed on the oil and protein concentration to assess the impact of biological replications. Statistics were generated and distribution plots were made for maturity, oil and protein concentrations, and genetic distinctness using R packages to characterize their variabilities. Trait correlations were also analyzed on the accessions having pairwise trait values. For example, there were 79 accessions having both days to maturity and protein data, and a correlation of these two trait data was made on these 79 accessions. The significant trait correlations were plotted using custom R scripts.

### 2.5. Developing Trait-Specific Core Subset

Based on the trait profiles generated from the characterization, we ranked the assayed accessions and selected a defined proportion of the assayed accessions with extreme (either lowest or highest) ranks, depending on the breeding target of the trait. For example, for germplasm with early maturity, the selection was aimed at the accessions with the fewest days to maturity. To search for germplasm with high oil or protein concentration, we selected accessions with the highest oil or protein concentration. In this paper, a trait-specific core subset size of 35 accessions was arbitrarily applied. To understand if a trait-specific core subset still genetically represents the whole collection, we also calculated the means of genetic distinctness both in the selected accessions and the whole collection and assessed the significance of their differences using a one-factor ANOVA test in R.

## 3. Results

### 3.1. Patterns of Variability

**Figure 1:**
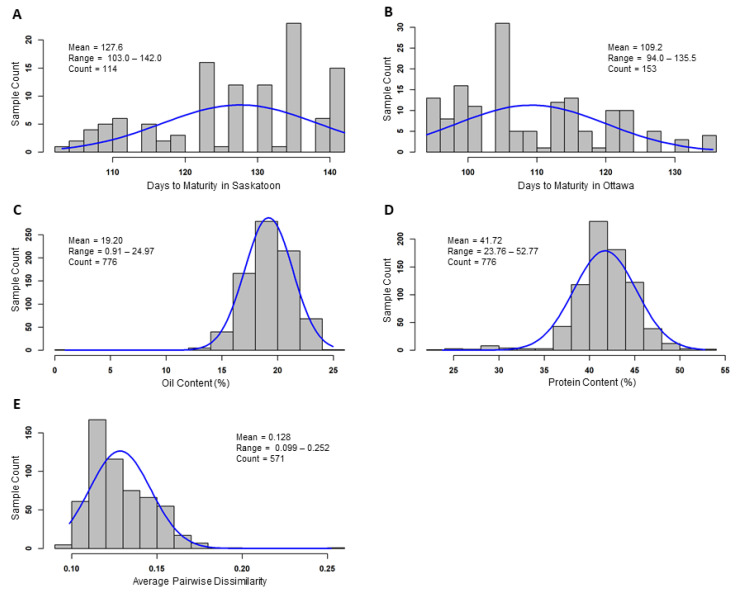
The distribution of observed values for the assayed soybean accessions in maturity, oil and protein concentration, and genetic distinctness. Panels (**A**,**B**) show the variabilities of soybean maturity observed in Saskatoon and Ottawa in 2018, respectively. Panels (**C**,**D**) illustrate the distributions of oil and protein concentration, respectively. Panel (**E**) displays the variability of genetic distinctness measured with average pairwise dissimilarity.

The field trial of 156 accessions revealed statistically significant (*p* < 0.05) differences in days to maturity among the tested accessions and between Ottawa and Saskatoon test sites, while replication effects were not statistically significant, based on the analysis of variance. Specifically, the days to maturity for the Saskatoon site ranged from 103 to 142 days with an average of 127.6 days (Figure 1A), while for the Ottawa site they ranged from 94 to 135.5 days with an average of 109.2 days (Figure 1B). When only the PGRC soybean accessions were considered, the range of the days to maturity in either test site remained the same as mentioned above, but the average showed 126.5 days in Saskatoon and 108.0 days in Ottawa or roughly one day earlier in either site (Table S1). The top five PGRC soybean accessions showing the earliest maturity in Saskatoon were CN39131, CN107883, CN107808, CN107648, and CN107526 from Canada, France, and Sweden, with the days to maturity ranging from 103.5 to 108 days. There were 13 PGRC soybean accessions originating from eight countries with the earliest maturity in Ottawa of 94 to 95 days (Table S1). Additional data on the other characters and field observations of soybean development collected at both test sites can be found in Tables S2 and S3.

The analysis of the 776 accessions for dry seed oil concentration revealed a significant difference in oil concentration among the assayed accessions, but not among the three biological replications, based on the analysis of variance. The estimated oil concentration per accession ranged from 0.9 to 25.0% with an average of 19.2% (Figure 1C). The top five PGRC soybean accessions with the highest oil concentration (of 23.8% or higher) were CN33273, CN107424, CN33279, and CN33261 from Canada and CN52634 from Russia (Table S1). Interestingly, there was one unique accession (CN45091) having the lowest oil concentration of 0.91% with a standard deviation of 0.01% for three biological replications. To verify the low oil concentration for CN45091, a retest with three new sets of seeds yielded a similar oil concentration, but a gas chromatography analysis of the three sample sets for total content of individual triglycerides following the method of Heydarian et al. [31] with modifications revealed a slightly higher oil concentration of 1.64%. Similarly, the protein analysis of the 776 accessions showed a significant difference in protein concentration among the assayed accessions, but not between the two biological replications. The estimated protein concentration per accession ranged widely from 23.8 to 52.8% with a mean of 41.7% (Figure 1D). The top five PGRC soybean accessions with the highest protein concentration were CN107869, CN107859, CN107872, CN107852, and CN107515 from China, Sweden, Poland, and France with 52.8 to 50.4% protein concentrations (Table S1).

The reanalysis of the published genetic distinctness values revealed a wide range of average pairwise dissimilarity values from 0.099 to 0.252 with an average of 0.128 and standard deviation of 0.018 (Figure 1E). Such variability remained the same if only the 541 PGRC soybean accessions were considered. There were nine accessions originating from seven countries displaying the highest average pairwise dissimilarity values of 0.252 to 0.171 (Table S1).

### 3.2. Patterns of Trait Correlation

**Figure 2:**
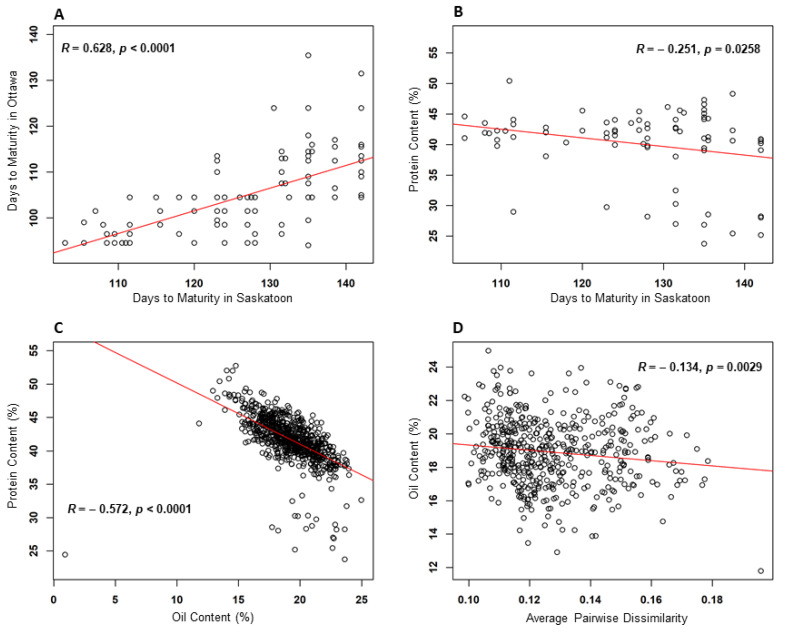
Four significant trait correlations detected among maturity, oil and protein concentration, and genetic distinctness of the assayed soybean accessions.

The pairwise correlation analysis among maturity, oil, protein, and genetic distinctness revealed only four (out of 10) pairwise significant correlations (as shown in Figure 2). The most significant correlations were between the maturity estimates at the two locations (*R* = 0.628, *p* < 0.0001) (Figure 2A) and between oil and protein concentration (*R* = −0.572, *p* < 0.0001) (Figure 2C). The estimated days to maturity at the Saskatoon site were negatively correlated with protein concentration (*R* = −0.251, *p* = 0.0258) (Figure 2B). The oil concentration estimates were negatively correlated with the values of genetic distinctness (*R* = −0.134, *p* = 0.0029) (Figure 2D). Six non-significant (*p* > 0.05) pairwise correlations are shown in Figure S1.

### 3.3. Trait-Specific Core Subset

The germplasm characterizations generated trait profiles that allowed for the identification of germplasm accessions with extreme trait values. Based on these trait profiles, we identified 35 accessions from the PGRC soybean collection with the earliest maturity in Saskatoon and Ottawa (Table 1), together with the highest oil and protein concentration (Table 2). 

The core subset for early maturity in Saskatoon consisted of 35 accessions originating from more than 10 countries and had days to maturity ranging from 103 to 126 days and averaging 115.7 days (Table 1). This subset matured 12 days earlier on average than the 114 accessions that reached maturity, which had a mean of 127.6 days. This difference was highly significant statistically. The core subset for early maturity in Ottawa consisted of 35 accessions which originated from more than 13 countries and had an average of 96.7 days to maturity and ranged from 94 to 102 days to maturity (Table 1). Similarly, this subset matured 12.5 days earlier on average than the 153 assayed accessions with a mean of 109.2 days to maturity. This difference was also highly significant statistically. Interestingly, these two maturity core subsets shared 24 accessions, confirming the consistency of trait performances over the two sites.

The core subset for high oil concentration consisted of 35 accessions that originated from six countries (largely from Canada) and showed the highest oil concentration ranging from 25.0 to 22.7% with an average of 23.2%. This subset significantly increased the total oil concentration, which was 4.0% higher than the assayed 766 accessions (19.2%). The core subset for high protein concentration consisted of 35 accessions that originated from more than 13 countries and displayed the highest protein concentrations ranging from 52.8 to 46.7% with an average of 48.4%. This core subset had a protein concentration 6.7% higher than that of the 776 assayed accessions (41.7%). Consistent with the negative correlations between the two traits, these two subsets did not have any accessions in common. 

Further analysis of the differences in the means of average pairwise dissimilarity between a trait-specific core subset and all of the assayed accessions did not reveal any significance for any core subset. The mean and standard deviation of average pairwise dissimilarity were 0.1277 and 0.0155 for the core subset for early maturity in Saskatoon; 0.1273 and 0.0148 for the core subset for early maturity in Ottawa; 0.1259 and 0.0193 for the oil core subset; and 0.1302 and 0.0144 for the protein core subset. None of these four means was statistically significant from those (0.1284 and 0.0180) of the 531 assayed accessions (Table S1), based on the one-factor ANOVA analyses. 

## 4. Discussion

These characterization efforts have revealed a wide range of variations present in maturity, oil and protein concentration, and genetic distinctness among the soybean accessions conserved in Plant Gene Resources of Canada. These trait variabilities have allowed for the development of the four core subsets of soybean germplasm, each specifically targeted for early maturity and high oil and protein concentration. These new core subsets will be useful for the management and utilization of the conserved soybean germplasm. 

While our characterizations were informative, the trait analyses varied greatly in the number of accessions assayed, so the amount of information on each trait also varied. For example, the field assessment of maturity was limited to 116 PGRC soybean accessions and two locations with only two one-row replications, while the oil and protein analyses were conducted on 776 accessions with two to three biological replications. These variations resulted from limited research resources. Ideally, more accessions should be analyzed in more locations with more replications for multiple years, especially for growth traits. Further quality analyses such as oil and protein compositions may be pursued to generate more informative quality trait profiles. 

To our knowledge, few studies have characterized soybean maturity in Saskatchewan [18]. The revealed soybean maturity profile in Saskatoon (Table S1) was consistent with the report by MacMillan and Gulden [19] with 106 to 115 days to maturity in the Northern Great Plains. The observed soybean maturity data in Ottawa was compatible with those reported by Ort et al. [20] in Ontario. Our initial analyses of oil and protein concentration potential revealed wider ranges of variation than those reported in the recent meta-analyses of US soybean seed yield and composition [32]. Additionally, the averaged estimates of oil and protein concentration were much higher than those reported for soybeans grown in the Northern Great Plains [19]. These comparisons suggested that the PGRC soybean collection can contribute to the search for useful germplasm with higher oil or protein concentration for soybean breeding. However, it is worth noting that the oil and protein profiles of PGRC soybean germplasm may vary if these accessions are grown in different environments, as the meta-analysis by Rotundo and Westgate [33] showed that environment (year and location) strongly affected soybean protein and oil concentration.

The PGRC soybean collection is small in size when compared to the collection of 33,000 accessions at the Chinese Crop Germplasm Resources Bank (CCGRB) [34] and the USDA-ARS soybean collection with 20,000 accessions [35], but it consists largely of the short-season germplasm originating from more than 30 countries, mainly from Europe and Asia. The acquired oil and protein profiles were compatible with the ranges of 14.5 to 23.0% for oil and 33.1 to 49.2% for protein in the early survey of 60 USDA-ARS soybean accessions [36]. Specifically for protein, the acquired profile ranging from 23.8 to 52.8% and averaging 41.7% was also compatible with those reported on worldwide conserved soybean accessions (as reviewed by Guo et al. [34]) and matched well with the variability from 29.3 to 52.9% with a mean of 44.3% reported among 21,050 assayed CCGRB soybean accessions [37]. Thus, the PGRC soybean collection itself could serve as a proxy core set to explore high protein germplasm for soybean breeding.

The trait-specific core subsets we developed showed the benefits of acquiring soybean germplasm with the earliest maturity and highest oil and protein concentration when compared with those trait profiles of all of the assayed accessions. For example, the two maturity subsets displayed maturity 12 days earlier on average than all of the assayed accessions. The protein subset had a protein concentration 6.7% higher than that of the 776 assayed accessions. These four core subsets did not differ significantly in genetic distinctness from the whole soybean collection, suggesting that the selected accessions still possess genetic diversity similar to the whole collection. This similarity in genetic diversity is partly expected, given the weak correlations of the assayed traits with genetic distinctness and the number of countries that the selected 35 accessions represented.

It is worth mentioning more regarding the developed core subsets. First, these core subsets focused on the PGRC soybean accessions and did not include those varieties and breeding lines assayed from Canadian soybean breeding programs. The related information on those exclusive varieties and breeding lines can be found in Table S1. For example, the variety ‘AAC Edward’ took 107 days to reach maturity at Saskatoon (Table S1), indicating that it is adapted to the region. Second, we arbitrarily defined a trait-specific core subset size of 35 accessions, as we reasoned this set should have roughly a 5% representation of the PGRC soybean collection with seeds available for distribution. However, an expansion of the subset with more accessions is possible and could be made from Table S1. Third, the core subsets were developed on the revealed trait variability from this characterization only and could be modified if more informative characterization is made in the future.

Our analyses also revealed four significant correlations among the assayed traits. The maturity measures of the assayed soybean accessions between the two test sites were significantly different in means but were highly correlated (Figure 2A). The difference in the average days to maturity in Saskatoon and Ottawa (127.6 and 109.2 days, respectively) may largely reflect the differences in climate and photoperiod between the two locations. The observed correlation in maturity between the two locations was consistent with the sharing of the 24 accessions between the two maturity core subsets. A significant negative correlation between seed oil and protein concentration (Figure 2C) was also observed in this study. Such correlation has been well documented [38,39] and it has been difficult for breeders to develop high-oil soybean genotypes while retaining a high level of protein. More interestingly, protein concentration was significantly correlated with maturity in Saskatoon (Figure 2B), but not with maturity at the Ottawa site (Figure S1), indicating that the earlier lines that were capable of maturing were also capable of storing seed protein. Additionally, genetic distinctness was significantly associated with the seed oil concentration only (Figure 2D), not with the protein concentration, or the maturity data. This finding implies that the extent of genetic diversity may not always be associated with specific traits of a breeding target.

The results presented here have some practical implications. First, the findings of the trait variability and correlation are useful for further soybean research, particularly for the adaptation to higher latitudes. For example, further research is required to validate if the lines that were capable of early maturing were also capable of storing seed protein on the Canadian Prairies. It is also desirable to explore the relationships between early maturity, oil and protein concentration, and yield in the Canadian Prairies. Second, the characterization efforts have generated the first large set of characterization data of the PGRC soybean germplasm in maturity, oil and protein concentration, and genetic distinctness. This data can be integrated into the genebank database such as GRIN-CA for public access. The data could also be useful for setting up management priorities to conserve the germplasm and identifying backup accessions for safety backup. Third, the developed core subsets will enhance the use of the PGRC soybean collection by Canadian soybean breeders. The maturity core subsets showed the PGRC soybean collection had accessions four days earlier to maturity than the current variety ‘AAC Edward’ at the Saskatoon site, suggesting that the PGRC soybean collection can contribute to the expansion of soybean production on the Canadian Prairies. Some selected PGRC soybean accessions can be explored to enhance oil concentration. Additionally, there is one unique accession (CN45091) with the lowest oil concentration of 0.91 to 1.64% and low protein concentration of 24.5% (Table S1). Similarly, the protein core subset has accessions with protein concentrations up to 52.8%, which may warrant incorporation into breeding programs. These core subsets of germplasm can be acquired for research and breeding via PGRC germplasm requests (https://pgrc-rpc.agr.gc.ca/gringlobal/search; accessed on 19 November 2022).

## 5. Conclusions

The characterization of soybean germplasm has revealed a wide range of variations present in maturity, oil and protein concentration, and genetic distinctness among the soybean accessions conserved in Plant Gene Resources of Canada. The revealed variability helped to identify four core subsets of 35 PGRC soybean accessions, each specifically targeted for the earliest maturity grown in Saskatoon and Ottawa and for the highest oil and protein concentration. These core subsets did not differ significantly in genetic distinctness from the whole PGRC soybean collection. These findings are useful, particularly for the management and utilization of conserved soybean germplasm.

## Figures and Tables

**Table 1:** The list of PGRC soybean accessions for maturity core subsets developed for growing in Saskatoon and Ottawa, along with their accession information and trait values (mean and standard deviation (SD)).

				DTM Saskatoon						DTM Ottawa	
Key	Accession	Origin	Description	Mean	SD	Key	Accession	Origin	Description	Mean	SD
236	CN39131	CAN	X763/3/B	103.0	1.4	595	CN107602	ROU	Herb 620	94	na
671	CN107808	FRA	Grignon 1	105.5	0.7	36	CN31692 *	DEU	No. 409	94.5	0.7
746	CN107883	FRA	Grignon 39	105.5	0.7	211	CN39087 *	CAN	X702/3/3	94.5	0.7
519	CN107526	SWE	748/5	108.0	0.0	236	CN39131 *	CAN	X763/3/B	94.5	0.7
641	CN107648	UNK	PI 840/2/7	108.0	0.0	320	CN52628 *	SWE	PGR 27203	94.5	0.7
320	CN52628	SWE	PGR 27203	108.5	0.7	457	CN107464 *	GBR	PI 152573	94.5	0.7
640	CN107647	UNK	PI 827/4/23/46	108.5	0.7	462	CN107469	CAN	Manitoba Brown	94.5	0.7
528	CN107535	SWE	634/20/4/29	109.5	2.1	508	CN107515 *	FRA	Geant Vert	94.5	0.7
590	CN107597	JPN	(Shinsei)	109.5	2.1	556	CN107563	HUN	Wielnska Brunatna	94.5	0.7
649	CN107656	SWE	Fiskeby V	109.5	2.1	632	CN107639 *	POL	Bydgoska 074	94.5	0.7
211	CN39087	CAN	X702/3/3	110.5	2.1	649	CN107656 *	SWE	Fiskeby V	94.5	0.7
508	CN107515	FRA	Geant Vert	111.0	0.0	694	CN107831 *	POL	Zlotka	94.5	0.7
165	CN35918	RUS	Saliut 216	111.5	0.7	746	CN107883 *	FRA	Grignon 39	94.5	0.7
479	CN107486	NLD	No. 709	111.5	0.7	463	CN107470	BEL	B/24	96.5	3.5
520	CN107527	SWE	748/7	111.5	0.7	514	CN107521 *	SWE	698/3/5	96.5	2.1
632	CN107639	POL	Bydgoska 074	111.5	0.7	520	CN107527 *	SWE	748/7	96.5	3.5
317	CN51844	CAN	KG20	115.5	6.4	528	CN107535 *	SWE	634/20/4/29	96.5	3.5
509	CN107516	FRA	Rouest 13 Al 2	115.5	4.9	590	CN107597 *	JPN	(Shinsei)	96.5	2.1
513	CN107520	SWE	698/1/1	115.5	0.7	640	CN107647 *	UNK	PI 827/4/23/46	96.5	3.5
583	CN107590	RUS	Amurskaja 310	115.5	6.4	690	CN107827	RUS	Salut 216	96.5	2.1
514	CN107521	SWE	698/3/5	118.0	2.8	161	CN35757	UKR	Terezikskaja 24	98.5	0.7
494	CN107501	FRA	Starachramiskaya	120.0	0.0	180	CN36143	CAN	Maple Presto H.P.	98.5	0.7
694	CN107831	POL	Zlotka	120.0	0.0	475	CN107482	NLD	No. 47	98.5	0.7
540	CN107547	POL	Bydgoska 052	123.0	4.2	479	CN107486 *	NLD	No. 709	98.5	0.7
591	CN107598	ROU	F. 166	123.0	4.2	509	CN107516 *	FRA	Rouest 13 Al 2	98.5	0.7
636	CN107643	POL	Zolta Przebedowska	123.0	4.2	519	CN107526 *	SWE	748/5	98.5	0.7
652	CN107659	UNK	Heimkraft 1	123.0	4.2	542	CN107549 *	DEU	C 7/58	98.5	0.7
36	CN31692	DEU	No. 409	124.0	5.7	641	CN107648 *	UNK	PI 840/2/7	98.5	0.7
300	CN42821	CAN	Maple Ridge	124.0	5.7	652	CN107659 *	UNK	Heimkraft 1	98.5	0.7
328	CN52639	RUS	Aurora	124.0	5.7	685	CN107822 *	DEU	Dieckman Black	98.5	0.7
457	CN107464	GBR	PI 152573	124.0	5.7	718	CN107855	SWE	754/5	98.5	0.7
524	CN107531	SWE	751/3	124.0	5.7	747	CN107884	SWE	291/1/2	98.5	0.7
542	CN107549	DEU	C 7/58	124.0	5.7	671	CN107808 *	FRA	Grignon 1	99	na
685	CN107822	DEU	Dieckman Black	124.0	5.7	529	CN107536	SWE	706/4/1	99.5	6.4
635	CN107642	POL	Zlocista	126.0	0.0	317	CN51844 *	CAN	KG20	101.5	3.5
	Average			115.7	2.7		Average			96.7	1.4

Note that Key is the same sample labelling as in Table S1. The accessions with CN label were from the PGRC soybean collection. The country of origin is given in the ISO 3166 code; UNK is for unknown. DTM is days to maturity and na is not available. The star * following accession label for the Ottawa subset indicates a shared accession with the Saskatoon subset.

**Table 2:** The list of PGRC soybean accessions for oil and protein core subsets, along with their accession information and trait values (mean and standard deviation (SD)).

				Oil %						Protein %	
Key	Accession	Origin	Description	Mean	SD	Key	Accession	Origin	Description	Mean	SD
84	CN33273	CAN	Beechwood	24.97	0.91	732	CN107869	CHN	(Suen wu dah bai mee)	52.77	1.28
423	CN107424	CAN	AC Harmony	23.97	0.47	722	CN107859	SWE	Ugra Saja	52.07	2.21
89	CN33279	CAN	Starbuck	23.96	0.05	735	CN107872	POL	Bydgoska 072	51.99	1.50
73	CN33261	CAN	Evans	23.81	0.41	715	CN107852	SWE	737/1	50.82	0.12
323	CN52634	RUS	CH20951	23.77	0.24	508	CN107515	FRA	Geant Vert	50.42	0.05
296	CN42388	CAN	A1937	23.77	1.01	205	CN39080	CAN	X691/19/2	49.37	1.12
824	CN120155	CAN	Tala	23.67	0.54	127	CN35344	KOR	KAS362/7	49.02	0.11
344	CN52735	CAN	OT93/28	23.63	0.36	731	CN107868	FRA	Ronset 4	48.88	0.20
27	CN30633	HUN	Iregi Korona	23.63	0.62	201	CN39073	CAN	X690/1/4	48.78	0.08
181	CN36214	CHN	Gang 6634/7/2	23.62	0.42	708	CN107845	BEL	B/19	48.75	1.34
343	CN52734	CAN	OT93/26	23.59	0.79	470	CN107477	UNK	V/6	48.63	1.67
324	CN52635	RUS	CH20950	23.52	0.19	204	CN39079	CAN	X691/18/2	48.54	1.32
288	CN39196	CAN	X1568B	23.28	0.12	638	CN107645	JPN	Sakamotowase	48.46	0.95
388	CN107385	CAN	Maple Belle	23.10	0.17	501	CN107508	FRA	Mandchourie	48.41	0.05
417	CN107418	CAN	B0501	23.09	0.52	176	CN36138	CAN	CZ/13/2/4	48.33	0.52
197	CN39065	CAN	K160/10/3/1/1/2	23.08	0.41	145	CN35371	KOR	KAS643/1	48.30	1.27
72	CN33260	CAN	Merit	23.08	0.59	645	CN107652	USA	Sioux	48.08	1.74
819	CN120150	UKR	UKR-2005 23-2	23.03	0.40	157	CN35386	KOR	KAS663/1	47.96	3.07
66	CN33253	CAN	Morsoy	22.96	0.36	485	CN107492	DEU	Strain No. 42	47.84	0.31
330	CN52641	RUS	Vzlyot	22.96	0.48	200	CN39072	CAN	X690/1/3	47.81	0.01
178	CN36141	CAN	K357/1/8/2/5	22.91	0.18	608	CN107615	DEU	Hercumft	47.68	1.55
414	CN107415	CAN	Alta	22.89	0.15	637	CN107644	POL	Zolta z Zolna	47.59	0.85
293	CN42379	CAN	Gesto	22.89	1.01	141	CN35364	KOR	KAS629/1	47.53	1.48
291	CN39217	CAN	X1592	22.86	0.47	492	CN107499	DEU	Strain No. 164	47.47	1.80
165	CN35918	RUS	Saliut 216	22.85	0.83	503	CN107510	DEU	Bitterhof	47.40	1.97
346	CN52737	CAN	OT94/51	22.83	0.14	728	CN107865	YUG	Novosadska Rana	47.31	0.42
396	CN107397	CAN	PS 55	22.82	0.45	643	CN107650	CHN	Salut 216 China	47.25	1.44
292	CN42220	CAN	Corsoy 79	22.75	0.25	621	CN107628	CHN	Suen wu dah bai mee	47.09	1.37
54	CN32631	CHE	PGR 4172	22.74	1.10	468	CN107475	NLD	J/5A	47.05	1.66
391	CN107391	CAN	HS 3078	22.72	0.62	237	CN39134	CAN	X779/12/B	47.02	0.39
362	CN107359	CAN	Frima	22.72	0.52	206	CN39081	CAN	X693/5/1	47.01	0.06
345	CN52736	CAN	OT94/49	22.70	0.13	228	CN39116	CAN	X735/1/B	46.83	0.62
823	CN120154	CAN	Niagra	22.69	1.66	164	CN35917	RUS	Ussurijskaja 477	46.82	0.76
210	CN39086	CAN	X702/3/2	22.68	0.54	710	CN107847	BEL	B/22	46.74	1.16
79	CN33268	CAN	Crest	22.67	0.75	504	CN107511	FRA	Jaune De Desme	46.68	0.27
	Average			23.21	0.51		Average			48.36	0.99

Note that Key is the same sample labelling as in Table S1. The accessions with CN label were from the PGRC soybean collection. The country of origin is given in the ISO 3166 code; UNK is for unknown. These two subsets do not have any accessions in common.

## Data Availability

The data presented in this study are available in Supplementary Materials.

## Supplementary Materials

The following supporting information can be downloaded at: https://doi.org/10.6084/m9.figshare.21587142, Table S1: The list of the 824 assayed accessions of the PGRC soybean collection, 30 lines and 10 checks from the AAFC Ottawa soybean breeding program, along with their related information and observed trait values (mean and standard deviation (SD)) in maturity, oil and protein concentration, and genetic distinctness (APD), Table S2: Additional phenotypic data collected from the maturity field trials at the Saskatoon and Ottawa test locations in 2018, Table S3: Additional field observations of soybean development from seeding to maturity following the Fehr and Caviness scale at the Saskatoon and Ottawa test locations in 2018, Figure S1: Six non-significant (*p* > 0.05) trait correlations observed among maturity, oil and protein concentration, and genetic distinctness of the assayed soybean accessions.
